# Antimicrobial Resistance in Romania: Updates on Gram-Negative ESCAPE Pathogens in the Clinical, Veterinary, and Aquatic Sectors

**DOI:** 10.3390/ijms24097892

**Published:** 2023-04-26

**Authors:** Ilda Czobor Barbu, Irina Gheorghe-Barbu, Georgiana Alexandra Grigore, Corneliu Ovidiu Vrancianu, Mariana Carmen Chifiriuc

**Affiliations:** 1 Microbiology-Immunology Department, Faculty of Biology, University of Bucharest, 050095 Bucharest, Romania; 2 The Research Institute of the University of Bucharest, 050095 Bucharest, Romania; 3 National Institute of Research and Development for Biological Sciences, 060031 Bucharest, Romania; 4 Academy of Romanian Scientists, 050044 Bucharest, Romania; 5 Romanian Academy, 010071 Bucharest, Romania

**Keywords:** Gram-negative ESCAPE pathogens, resistance, intra-hospital infections, community-acquired, aquatic resistome

## Abstract

Multidrug-resistant Gram-negative bacteria such as *Acinetobacter baumannii*, *Pseudomonas aeruginosa*, and members of the Enterobacterales order are a challenging multi-sectorial and global threat, being listed by the WHO in the priority list of pathogens requiring the urgent discovery and development of therapeutic strategies. We present here an overview of the antibiotic resistance profiles and epidemiology of Gram-negative pathogens listed in the ESCAPE group circulating in Romania. The review starts with a discussion of the mechanisms and clinical significance of Gram-negative bacteria, the most frequent genetic determinants of resistance, and then summarizes and discusses the epidemiological studies reported for *A. baumannii*, *P. aeruginosa*, and Enterobacterales-resistant strains circulating in Romania, both in hospital and veterinary settings and mirrored in the aquatic environment. The Romanian landscape of Gram-negative pathogens included in the ESCAPE list reveals that all significant, clinically relevant, globally spread antibiotic resistance genes and carrying platforms are well established in different geographical areas of Romania and have already been disseminated beyond clinical settings.

## 1. Introduction

Gram-negative bacteria represent one of the most significant pathogens involved in public health issues. Among them, members of the families Enterobacterales, Moraxellales, and Pseudomonadales have a major clinical significance, being key Gram-negative pathogens listed in the ESKAPE (*Enterococcus faecium*, *Staphylococcus aureus*, *Klebsiella pneumoniae*, *Acinetobacter baumannii*, *Pseudomonas aeruginosa*, and *Enterobacter* species) or later designated ESCAPE group (*E. faecium*, *S. aureus*, *Clostridioides difficile*, *A. baumannii*, *P. aeruginosa*, Enterobacterales), as well as in the WHO priority pathogens list for R&D of new antibiotics in Priority 1: CRITICAL list (namely carbapenem-resistant *A. baumannii*, carbapenem-resistant *P. aeruginosa*, and carbapenem-resistant, extended-spectrum β-lactamase (ESBL)-producing Enterobacterales) [1]. The Centers for Disease Control and Prevention (CDC) described the dramatic increase of antibiotic resistance (AR) during the last decades as one of the most critical threats to public health, with very few effective antimicrobials left, either novel or old molecules [2,3], therefore requiring concerted research and management efforts [4].

Gram-negative bacteria possess multiple AR mechanisms towards all antibiotic classes: besides intrinsic resistance (mainly due to their outer membrane, efflux pumps, and extra wall structures), they may acquire resistance via chromosomal mutations and horizontal gene transfer (HGT) [5]. Chromosomal mutations may alter some critical enzymes (for example, mutations in genes encoding DNA gyrases and topoisomerases lead to quinolone resistance) and regulatory proteins (as mutations in the *mgrB* small regulatory protein in *K. pneumoniae* lead to colistin resistance) that may be involved in the loss, downregulation, or alteration of porins or may lead to an increased expression level of efflux pumps. Nevertheless, the main mechanisms involved in AR in Gram-negative bacteria are represented by hydrolytic enzymes, especially β-lactamases, acquired via HGT [5].

In this context, we aimed to bring together all the available epidemiological studies reported for *A. baumannii*, *P. aeruginosa*, and Enterobacterales-resistant strains circulating in Romania in different environments (clinical, community, veterinary settings, wastewater, and surface water networks) in order to take a step further to illuminate the extent of the dissemination of antibiotic resistance genes (ARGs) in our country, taking into account that in Romania there has not yet been implemented a national action plan to combat the dissemination of AR.

## 2. Main Mechanisms of AR in Gram-Negative Pathogens

### 2.1. β-Lactam Resistance

Resistance to β-lactam antibiotics (penicillins, cephalosporins, carbapenems, monobactams, and β-lactamase inhibitors) is conferred by the production of antibiotic-modifying enzymes, efflux pumps, porins, protection of the antibiotic target, or biofilm production [6,7,8,9]. However, the primary mechanism of β-lactam resistance in Gram-negative bacilli is represented by the production of β-lactamases (serine-β-lactamases—Ambler classes A, C, and D, and metallo-β-lactamases or MBL—Ambler class B) [10,11,12].

Class A β-lactamases (except for *Klebsiella pneumoniae* carbapenemase—KPC) hydrolyze penicillins and cephalosporins more efficiently than carbapenems and are inhibited by clavulanic acid [13]. It includes narrow spectrum, or ESBLs, inhibited by clavulanic acid or tazobactam, and they confer penicillin, cephalosporins, monobactams, and carbapenem resistance [14] (Table 1). In addition, it is well known that ESBLs play an essential role in resistance against later-generation cephalosporins such as cefotaxime, ceftazidime, and cefepime [15]. Class B β-lactamases include metallo-β-lactamases (MBLs), broad-spectrum enzymes that require zinc or another heavy metal for hydrolysis β-lactam antibiotics except for monobactams [13]. Class C β-lactamases, also known as AmpC β-lactamases, confer resistance to cephamycins, penicillins, cephalosporins, and β-lactamase inhibitors [13], which can be: (a) inducible resistance via chromosomally encoded *ampC* genes (e.g., *Enterobacter cloacae*, *Serratia marcescens*, *Citrobacter freundii*, *P. aeruginosa*), (b) non-inducible chromosomal resistance due to promoter and/or attenuator mutations (e.g., *Escherichia coli, Shigella* spp., *A. baumannii*), or (c) plasmid-mediated resistance (e.g., *Klebsiella pneumoniae*, *E. coli, Salmonella* spp.). Class D β-lactamases include oxacillinases (OXAs) or carbapenem-hydrolyzing class D β-lactamases (CHDLs) that are serine β-lactamases that hydrolyze all β-lactam antibiotics and are not inhibited by clavulanic acid, sulbactam, or tazobactam [16].

AR caused by β-lactamase production may significantly increase when interacting with outer membrane proteins (OMPs) or efflux pumps. OMPs are monomeric or trimeric porins that mediate the diffusion of small molecules into or out of the periplasmic space of Gram-negative bacteria and can be classified as non-specific or specific according to their activity [17]. *Efflux* pumps actively control the intracellular concentration of antibiotic molecules with different chemical structures and are involved in multidrug resistance (MDR) mechanisms. The MDR efflux pumps belong to the ATP-binding cassette (ABC), major facilitator superfamily (MFS), small multidrug resistance (SMR), multidrug and toxic compound extrusion (MATE), proteobacterial antimicrobial compound efflux (PACE), and resistance-nodulation-cell division (RND) superfamilies and are the focus of current research [18].

In *A. baumannii*, β-lactam resistance is encoded by transferable β-lactamases from class A [19], class B [20,21], chromosomal class C β-lactamases (ADC), and chromosomal or plasmidial CHDLs (class D β-lactamases) (Table 1). The most characteristic β-lactamases in *A. baumannii* are represented by CHDLs: OXA-23, OXA-24/-40, OXA-58, OXA-143, and OXA-235 [14] (Table 1). The first OXA enzyme with carbapenemase activity in *A. baumannii,* ARI-1, was identified in a clinical strain isolated in Scotland and was subsequently renamed OXA-23 [22] (Table 1). This enzyme is disseminated worldwide due to its association with several transposons (Tn*2006*, Tn*2007*, Tn*2009*, Tn*2008*, and Tn*2008B)* [23].

In *P. aeruginosa,* there were described ESBLs from class A, represented mainly by VEB (Vietnamese extended-spectrum β-lactamase) and PER (*Pseudomonas* extended resistance), but also CTX-M (Cefotaxime first isolated in Munich), TEM (for Temoneira patient’s name), SHV (Sulfydryl Variable enzyme), and BEL (Belgium ESBLs) families [24,25,26,27]. Class A carbapenemases encountered in *P. aeruginosa* are represented by GES (Guyana Extended Spectrum β-lactamase) and KPC enzymes [28] (Table 1). Acquired MBLs in *P. aeruginosa* include the VIM (Verona imipenemase), IMP (active-on-imipenem), SPM (Saõ Paolo metallo-β-lactamase), SIM (Seoul imipenemase), FIM (Florence imipenemase), AIM (Australian imipenemase), and DIM (Dutch imipenemase) enzymes [29,30]. Class D comprises OXA enzymes, the name being derived from their preference for oxacillin and cloxacillin hydrolysis. They are mostly narrow-spectrum β-lactamases that confer resistance to cefotaxime or ceftazidime, with some OXA β-lactamases linked to resistance and/or reduced susceptibility to cefepime and/or aztreonam [30,31,32,33].

In Enterobacterales, the main mechanisms of β-lactam resistance are represented by the expression of class A ESBLs (mainly CTX-M and variants of TEM, SHV), classes A, B, and D carbapenemases [KPC, New Delhi MBL (NDM), OXA-48, IMP, VIM), or class C AmpC chromosomal or plasmidial enzymes [CMY (confer cephamycin resistance), FOX (cefoxitin resistance), MOX (moxalactam resistance), LAT (latamoxef resistance), ACC (Ambler class C), ACT (AmpC type), MIR-1 (Miriam Hospital in Providence), DHA (Dhahran hospital in Saudi Arabia)] [34,35].

### 2.2. Resistance to Other Antibiotic Classes

#### 2.2.1. Aminoglycoside Resistance

Aminoglycoside resistance in Gram-negative bacilli is mainly encoded by enzymatic resistance mechanisms (by the production of aminoglycoside-modifying enzymes—AMEs) but also by alteration of the ribosome structure (by 16S rRNA methyltransferases) and limited antibiotic uptake (due to the loss of cell membrane permeability or hyperactivity of the efflux pumps) [36,37,38]. AMEs are classified as aminoglycoside phosphotransferases (APH), aminoglycoside acetyltransferases (AAC), aminoglycoside nucleotidyltransferases (ANT), and aminoglycoside adenylyltransferases (AAD) [36]. The most frequently detected AME-encoding genes in Gram-negative strains listed in the ESCAPE group are presented in Table 2.

#### 2.2.2. Tetracycline Resistance

Tetracyclines bind to the 30S ribosomal subunit and inhibit protein synthesis, stopping translation [39]. Several mechanisms cause tetracycline resistance: efflux dependent on ATP (Tet(A), Tet(B), Tet(C), Tet(D), and Tet(G)) [40,41,42], ribosomal protection proteins (Tet(M), Tet(O), and Tet(H)) [14,43], target modification, and enzymatic antibiotic inactivation (Tet(X)) [44,45].

**Table 1:** The most frequent transmissible β-lactamases and corresponding encoding genes in Gram-negative rods.

β-Lactamases Class (Ambler)	Resistance Mechanisms	Encoding Genes	Species	References
Class A	Narrow spectrum β-lactamases	*bla*_SCO-1_;	*A. baumannii*	[46,47,48,49,50,51,52,53,54]
		*bla*_PSE-1/CARB-2_; *bla*_PSE-4/CARB-1_;	*A. baumannii*, *P. aeruginosa*;	
		*bla*_CARB-3, -4_; *bla*_TEM-1, -2_, *bla*_SHV-1_;	*Enterobacterales*, *A. baumannii*, *P. aeruginosa*	
	Extended-spectrum β-lactamases (ESBLs)	*bla*_CARB-10_;	*A. baumannii*;	[24,55,56,57,58,59,60,61,62,63,64,65,66,67,68,69,70,71,72,73,74]
		*bla*_CTX-M-1, -2, -9, -14, -15, -27, -43, -92_; *bla*_GES-1, -11, -13_;	*Enterobacterales*, *A. baumannii*, *P. aeruginosa*;	
		*bla*_TEM-3, -4, -12, -21, -24, -42, -116_; *bla*_SHV-1, -2, -2a, -5, -12, -27, -41, -187_;	*Enterobacterales*, *A. baumannii*, *P. aeruginosa*;	
		*bla*_VEB-1, -2, -9_; bla_VEB-1_;	*A. baumannii*, *P. aeruginosa*;	
		*bla*_BEL-1, -2_;	*P. aeruginosa*, *Enterobacterales*;	
		*bla*_PER-1, -2, -7_;	*A. baumannii*, *P. aeruginosa*;	
	Carbapenemases	*bla*_KPC-2, -3, -4, -5, -10_;	*Enterobacterales*, *A. baumannii*, *P. aeruginosa*;	[68,75,76,77,78,79,80,81,82,83,84,85,86]
		*bla*_GES-2, -5, -6, -14, -15, -20_;	*P. aeruginosa*, *Enterobacterales*;	
		*bla*_GPC-1_;	*P. aeruginosa*;	
		*bla*_IMI-2_, *bla*_IMI-3_; *bla*_SHV-38_;	*Enterobacterales*	
Class B	Carbapenemases	*bla*_NDM-1, -2, -5_;_;_	*Enterobacterales*, *A. baumannii*, *P. aeruginosa*;	[20,87,88,89,90,91,92,93,94,95,96,97,98,99,100,101,102,103,104,105,106,107,108,109,110,111,112,113,114,115,116,117,118,119,120,121,122,123,124,125,126]
		*bla*_VIM-1, -2, -3, -11_;	*P. aeruginosa*, *Enterobacterales*, *A. baumannii*;	
		*bla*_GIMlike_; *bla*_SIM-1_;	*A. baumannii*, *P. aeruginosa*;	
		*bla*_IMP-1, -2, -4, -5, -6, -8, -9, -11, -14a, -19, -27, -51, -55_;	*Enterobacterales*, *A. baumannii*, *P. aeruginosa*;	
		*bla*_SPM-1_; *bla*_SMP-1_; *bla*_DIM_; *bla*_HMB-1_; *bla*_CAM_; *bla*_AIM-1_; *bla*_FIM-1_	*A. baumannii * *P. aeruginosa*	
Class C	Chromosomally-derived cephalosporinases	*bla*_ADC-25, -30, 73_; *bla*_AmpC-69, -70, -71_; *bla*_ADC-55, -67, -68, -196_	*A. baumannii*	[127,128,129,130,131,132,133,134,135,136]
		*bla*_CMY_, *bla*_FOX_, *bla*_ACT_, *bla*_DHA_, *bla*_ACC_;	*Enterobacterales*, *P. aeruginosa*;	[72,137,138]
Class D	Narrow spectrum	*bla*_OXA-4,_ *bla*_OXA-20,_ *bla*_OXA-47_;	*Enterobacterales*, *P. aeruginosa*, *A. baumannii*;	[139,140,141]
	ESBL	*bla*_OXA-1, -31_, *bla*_OXA-2, -161_, *bla*_OXA-5_, *bla*_OXA-10, -11, -7_; *bla*_OXA-18_, *bla*_OXA-31_; *bla*_OXA-45_, *bla*_OXA-46_;	*P. aeruginosa*, *Enterobacterales*;	[31,32,142,143,144,145]
	Carbapenem-hydrolyzing class D β-lactamases (CHDLs)	*bla*_OXA-51_; *bla*_OXA-23_; *bla*_OXA-24_; *bla*_OXA-58_; *bla*_OXA-143_; *bla*_OXA-235_; *bla*_OXA-198_; *bla*_OXA-48_ and *bla*_OXA-48-like_ (*bla*_OXA-181, -162, -232_);	*A. baumannii*; * P. aeruginosa*; *Enterobacterales*	[57,145,146,147,148,149,150,151,152,153,154,155,156,157,158,159]

#### 2.2.3. Fluoroquinolone Resistance

Quinolones or fluoroquinolones are broad-spectrum bactericidal antibiotics that disrupt DNA replication by inhibiting the activity of type II topoisomerase, DNA gyrase, and topoisomerase IV. Fluoroquinolones primarily affect gyrase activity, while toxicity against topoisomerase IV is secondary. Resistance to fluoroquinolones in Gram-negative bacilli is mediated by the underexpression of porins or the overexpression of cellular efflux pumps [160], mutations in gyrase and topoisomerase IV (*gyrA*, *gyrB*, and *parC*) [161], and plasmid-mediated quinolone resistance (PMQR), encoded by *qnr* and *oqx* genes [160,161,162,163,164,165,166,167] (Table 2).

**Table 2:** The most frequent aminoglycosides, tetracyclines, fluoroquinolones, and, polymyxins transmissible resistance genes in Gram-negative rods.

Antibiotic Class	Antibiotics	ARGs	Species	References
**Aminogly** **cosides** **AMEs**	gentamicin, sisomicin, fortimicin	*aac(3)-I * *a/b/c/e*	*A. baumannii*, *P. aeruginosa*, *E. coli*, *K. pneumoniae*, *K. aerogenes*, *E. cloacae*, *E. hormachei*	[14,36,78,168,169,170,171]
		*aac3-Ib-aac6′-Ib’*	*P. aeruginosa*	
	gentamicin, netilmicin, tobramycin, sisomicin, dibekacin	*aac(3)-II * *a/c/d/e*	*E. coli*, *K. pneumoniae*, *Pseudomonas* sp. *A. baumannii*, *E. cloacae complex*	[14,172,173,174,175,176,177]
		*aac(3)-IVa*	*A. baumannii*, *K. pneumoniae*, *E. coli*	[14,178]
	amikacin, gentamicin	*aac(6′)-Ia * */i/l/q/ae/af/ai/30/33/aacA43*	*P. aeruginosa*, *E. coli*, *E. cloacae*, *E. hormachei*,	[14,36,168,179,180,181,182,183,184,185]
		*aac(6′)-Ib * */aacA4*	*P. aeruginosa* * A. baumannii*, *K. pneumoniae*, *E. cloacae*, *E. coli*	
	kanamycin, tobramycin, amikacin, ciprofloxacin, norfloxacin	*aac(6′)-Ib-cr*	*E. coli*, *E. cloacae* complex, *K. pneumoniae*, *A. baumannii*, *P. aeruginosa*	[186]
		*aac(6′)-If * *aac(6′)-Ig/h/j/k/k/r/s/t/u/v/w/x/ad/ae//aa/ad/ * *aacA29*	*E. cloacae*, * A. baumannii * * P. aeruginosa*	[36,187,188]
	gentamicin	*aac(6′)-IIa/I31/32/c*	*P. aeruginosa*, *K. pneumoniae*, *A. baumannii*	[36,182,188]
	dibekacin, gentamicin, kanamycin, sisomicin, tobramycin	*ant(2″)-Ia*	*A. baumannii*, *K. pneumoniae*, *E. coli*	[168,189,190,191,192]
	spectinomycin, streptomycin	*ant(3”)-II*	*A. baumannii*	[14]
	amikacin, tobramycin isepamicin	*ant(4′)-IIa/b*	*P. aeruginosa*	[191,192,193]
	streptomycin	*aadA1*,*2*,*5*,*11*,*13*,*16*	*E. coli*, *K. pneumoniae*, *E. cloacae*, *P. aeruginosa*, *A. baumannii*	[14,168,194,195,196]
		*aadA6/aadA10*	*P. aeruginosa*	
	gentamincin b, kanamycin, neomycin, paromomycin, lividomycin, ribostamycin	*aph(3′)-Ia/b/c/*	*E. coli*, *K. pneumoniae*, *P. aeruginosa*, *A. baumannii*	[14,168,197]
	kanamycin, neomycin, butirosin, paromomycin, ribostamycin	*aph(3′)-IIa*	*E. asburiae*, *E. coli*, *A. baumannii*, *P. aeruginosa*, *K. pneumoniae*	[14,198]
	kanamycin, neomycin, paromomycin, ribostamycin, butirosin, amikacin, isepamycin	*aph(3′)-VIa*, *aph(3′)-VIb*	*A. baumannii*, *K. pneumoniae*	[14,36,199]
	hygromycin	*aph(4)-Ia*	*E. coli*, *K. pneumoniae*, *A. baumannii*	[14]
	streptomycin	*aph(6)-Id*	*A. baumannii*, *K. pneumoniae*, *E. coli*, *P. aeruginosa*	[168,200,201]
	streptomycin	*aph33ib*	*A. baumannii*	[168]
**Aminoglycosides: 16S** **rRNA methylase genes**	aminoglycosides (all)	*armA*	*A. baumannii * * P. aeruginosa*, *E. coli*, *K. pneumoniae*, *E. cloacae* complex	[100,192,202]
		*rmtA*, * rmtB*, *rmtC*, *rmtD*, *rmtE*, *rmtF*, *rmtG*, *rmtH*, *rmtF*	*P. aeruginosa* *A. baumannii* * P. aeruginosa*, *E. coli*, *K. pneumoniae*, *E. cloacae* complex	[14,193,202,203,204,205,206,207,208]
**Tetracycli** **nes**	doxycycline, tetracycline	*tet(A)*, *tet(G)*	*A. baumannii* *P. aeruginosa* *E. coli* *E. cloacae* complex *K. pneumoniae*	[14,41,42,43,45,209,210,211,212]
	minocycline	*tet(B)*,		
	tetracycline	*tet(C)*, *tet(D)*		
	oxytetracycline, tetracycline	*tet(H)*		
	tetracycline, doxycycline, minocycline	*tet(M)*, *tet(O)*		
	tetracyclines (all)	*tet(X)*		
**Fluoroqui** **nolones**	ciprofloxacin	*qnrA * *qnrB * *qnrS*	*K. pneumoniae*, *E. coli*, *E. cloacae* complex, *A. baumannii*, *P. aeruginosa*	[14,213]
**Polymyxins**	colistin	*mcr1-10*	*E. coli*, *K. pneumoniae*, *E. cloacae* complex, *P. aeruginosa*, *A. baumannii*	[214,215]

#### 2.2.4. Polymyxin Resistance

Polymyxins (colistin and polymyxin B) are last-resort antibiotics reintroduced into clinical practice to treat infections caused by MDR Gram-negative bacteria resistant to carbapenems. Resistance to polymixins occurs due to mutations in chromosomal genes involved in lipopolysaccharide structure (*mgrB, pmrABC, lpxACD*), plasmid-mediated LPS modification (*mcr* genes), or active efflux [216,217,218,219,220,221,222,223].

#### 2.2.5. Fosfomycin Resistance

Fosfomycin is a broad-spectrum antimicrobial agent that inhibits the final step of peptidoglycan biosynthesis. Resistance is attributed to the modification of transporters across the cytoplasmic membrane, amino acid substitution in the MurA active site, which decreases fosfomycin binding affinity, and the production of the fosfomycin-inactivating enzyme FosA [224].

#### 2.2.6. Antifolate Resistance

Antifolate antibiotics inhibit purine metabolism and DNA and RNA synthesis by interfering with folic acid biosynthesis. Sulfonamides bind dihydropteroate synthase (DHPS), a catalytic enzyme in the folic acid biosynthesis pathway, inhibiting dihydrofolic acid formation [225]. Trimethoprim is a dihydrofolate reductase (DHFR) inhibitor. Sulfonamides combined with trimethoprim (such as sulfamethoxazole) are well-known folate inhibitors. Resistance to sulfonamides occurs due to mutations of *the folP* gene encoding DHPS or the acquisition of alternative DHPS genes (*sul1, sul2, sul3, sul4*) with low affinity to sulfonamides [226]. Trimethoprim resistance is mediated by *dfr* genes encoding trimethoprim-resistant dihydrofolate reductases.

**Figure 1:**
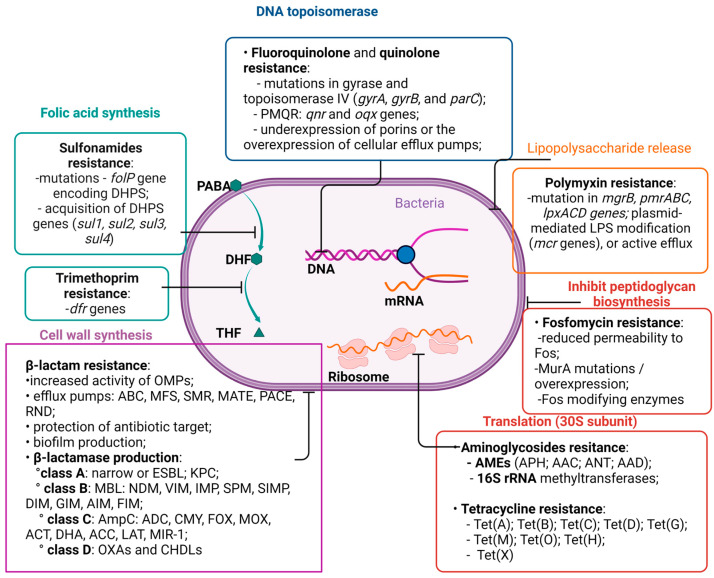
Schematic representation of mechanisms of actions and resistance to different antibiotic classes (Created with https://biorender.com/ accessed on 10 March 2023).

Gram-negative pathogens have developed resistance mechanisms to all antibiotics used for therapy (Figure 1).

### 2.3. Mobile Genetic Elements (MGEs)

MGEs [insertion sequences (IS), transposons (*Tn*), integrons, resistance islands, and plasmids] play a significant role in the evolution of prokaryotic genomes, conferring adaptative traits, including AR. Thus, MGEs can retain, capture, and disseminate the ARGs between bacterial strains or species responsible for the emergence of MDR [227,228].

#### 2.3.1. Insertion Sequences (IS)

IS are MGE that carry one or two transposase (tnp) genes and are responsible for the intracellular transportation of ARGs. ISs can be found across all prokaryotes, and their role in AR dissemination was well documented, not only by being able to mobilize ARGs but also by activating or inactivating specific genes in the bacterial chromosome, acting as promoters for silent ARGs or enhancing their expression, or, as in the case of certain enterobacteria, by inactivating certain porin-encoding genes or regulator genes [229,230]. In addition, two ISs can also form a composite transposon and sequester the genes between them [231].

In *A. baumannii,* the insertion of IS*Aba1*/*Aba2*/*Aba3*/*Aba4*/*Aba10* enhances the *bla*_OXA-51_, *bla*_OXA-23_, *bla*_OXA-58_ and *carO* genes expression, leading to carbapenem resistance, or with AmpC, leading to cephalosporin resistance [29,92,232,233,234,235,236,237,238,239]. In *P. aeruginosa*, the insertion of IS*Pa133*, IS*Pa1328*, IS*Pa46*, IS*Pa1635*, IS*Pa45*, IS*Pa26*, IS*Pa8*, and IS*Pre2*-like in the *oprD* porin-encoding gene confers carbapenem resistance [240,241,242,243,244,245,246]. In *K. pneumoniae* strains, the insertion of IS*1*,*5*,*26*,*903* in the *ompK36* porin-encoding gene confers cefoxitin resistance [247]. Colistin resistance emerges due to the insertion of IS*Aba11* in the *lpxA* or *lpxC* gene in *A. baumannii* [248] or IS*1Flike*, IS*5like*, IS*Kpn13*, IS*Kpn14* in the *mgrB* gene in *K. pneumoniae* [249,250].

The notorious IS*26* (or IS*6*) family is a well-known example of IS capable of sequestering and mobilizing ARGs. Members of the IS family are often found in arrays, in direct and/or inverted repeats, in MDR plasmids described in Gram-negative ESCAPE strains, and are able to capture virtually every ARG [251].

#### 2.3.2. Transposons (Tn)

Transposons are MGE that can harbor additional genes, including ARGs, besides transposases. The Tn3 family is the essential T*n* family involved in ARG transmission in Gram-negative and Gram-positive bacteria. *Tn3* usually contains β-lactamase resistance genes in Gram-negative bacteria; *Tn5* is associated with kanamycin, bleomycin, neomycin, and streptomycin; *Tn7* with trimethoprim, streptothricin, spectinomycin, and streptomycin; *Tn9* with chloramphenicol; and *Tn10* with tetracycline resistance [252,253,254].

#### 2.3.3. Integrons

MGE from the integrons group are located on bacterial chromosomes or plasmids. Four integron classes have been described in the nosocomial Gram-negative ESCAPE group, carrying ARGs responsible for β-lactams, aminoglycosides, and trimethoprim resistance [5]. In addition, different gene cassettes have been revealed: e.g., *bla_CARB-2_, aadA1, aadA2, aadB, dfrA1, dfrA7, dfrA1-gcuF, dfrA1- aadA1, dfr17-aadA5, dfr12-gcuF-aadA2, sat1* responsible for cephalosporins, aminoglycosides, and trimethoprim resistance in *A. baumannii* [255,256,257]; *aadA2, aadB, dfr17-aadA5, dfr12-gcuF- aadA2* associated with aminoglycosides and trimethoprim resistance in *P. aeruginosa* strains [258]; *bla*_CARB-2_, *bla*_GES-1_, *aadA, aadA1, aadB, dfrA1, dfrA7, dfrA1-gcuF, dfrA1-aadA1a, dfr17-aadA5, dfr12-gcuF-aadA2* with cephalosporins, trimethoprim and aminoglycosides resistance in *K. pneumoniae* [257,259]; *and aadA1, aadA2, aadA5 aadB, dfrA1, dfrA5, dfrA7 dfrA12, dfr14, dfrA17, dfrB2, dfrA1-gcuC, dfrA1-aadA1, dfr17-aadA5, dfr12-gcuF-aadA2, dfrA1-sat1-aadA1, dfrA1-sat2-aadA1, estX-sat2-aadA1, bla*_OXA-101_*-aac (6′)–Ib* with aminoglycosides, trimethoprim and cephalosporins resistance in *E. coli* [260]. Different integron structures were also found in strains from various sources. Soufi et al. [261] revealed the presence of different gene cassette arrays in *E. coli* isolates from poultry meat in Tunisia (*dfrA, aadA, sat-psp-aadA2-cmlA1- aadA1-qacH-IS440-sul3*), while Su et al. have shown the presence of class 1 and 2 integrons in *E. coli* from one river in South China [262].

#### 2.3.4. Genomic Resistance Islands

Genomic islands (GIs) represent clusters of genes of probable foreign origin, providing adaptative traits and representing a significant source of variation between bacterial strains. For example, GIs are responsible for forming different pathotypes of *E. coli* (uropathogenic—UPEC, enteropathogenic—EPEC, and enterohaemorrhagic—EHEC *E. coli*) [263]. GIs were associated with AR in *A. baumannii* epidemic clones, for which several *A. baumannii* Resistance Islands (AbaR) have been described [5], most of them in European clones I and II (Aba*R1*, Aba*R3*, Aba*R5*, Aba*R6*, Aba*R7*, Aba*R8*, Aba*R9*, and Aba*R10*) [264]. In *K. pneumoniae*, the GIE492 carries the *bla*_SHV-190_ gene, while in *E. cloacae*, the MIR17-GI carries *bla*_MIR17_ carbapenemase [265].

#### 2.3.5. Plasmids

Plasmids are the main shuttles for ARG dissemination. Complex MGEs may disseminate intra-species, inter-species, inter-genus, and even across more distantly related taxa. Plasmid dissemination was excellently described in the scientific literature [266,267]. In *A. baumannii*, a plasmid-based replicon typing scheme, currently containing 20 groups encoded GR1-GR20, has been proposed [268,269]. Different resistance plasmids carrying carbapenem resistance genes were reported in *P. aeruginosa* strains (e.g., IncP-1, IncP-2, IncP-6) [270,271,272]. In Enterobacterales, 26 different compatibility plasmid groups (Inc plasmids) have been described based on their compatibility with other plasmids in the same bacterial host [227]. The plasmid relaxase gene typing (PRaseT) allowed the classification into five relaxase clades designated HIα, HIβ, HIγ, HIδ, and HIɛ of IncHI1 and IncHI2 plasmids, to which IncX1–4 and ColE plasmids were added [273].

## 3. Clinical Significance of Antibiotic-Resistant Gram-Negative Pathogens

The MDR and virulence potential of *A. baumannii* are responsible for hospital and community-acquired infections [274]. *A. baumannii* is recognized as an opportunistic nosocomial pathogen, mainly in immunocompromised patients, and is frequently associated with therapeutic failures, especially during the COVID-19 pandemic. Several countries have reported that COVID-19 was associated with secondary MDR carbapenem-resistant *A. baumannii* (MDR CRAB) infections of the lower respiratory tract in intensive care unit (ICU) patients, emphasizing the importance of limiting the risk of co-infection and the dissemination of MDR CRAB strains in ICUs [275,276,277].

Carbapenem-resistant *P. aeruginosa* (CR-PA) is a major healthcare-associated pathogen worldwide [278]. *P. aeruginosa* is the primary cause of ventilator-associated pneumonia (VAP) in long-term acute care hospitals and hospital wards and the second most common cause of VAP in intensive care units. It is also the third most common cause of catheter-related urinary tract infections [279]. In *P. aeruginosa*, several mechanisms are responsible for carbapenem resistance. The first mechanism is the efflux pump, which is mediated by overexpression of the *MexAB-OprM* efflux pump, resulting in resistance to most β-lactam drugs except for imipenem. The second mechanism is the overproduction of AmpC beta-lactamase and the inactivation of the *OprD* outer membrane protein. This combination can lead to resistance to essentially all antipseudomonal β-lactams. Another resistance mechanism is the production of carbapenemases [280,281], which significantly alter the efficacy of commonly used antipseudomonal agents, including ceftazidime, cefepime, and piperacillin-tazobactam, as well as the newly introduced β-lactam/β-lactamase inhibitor combinations such as ceftolozane-tazobactam, imipenem-relebactam, and ceftazidime-avibactam. The carbapenem resistance determinants carried by *P. aeruginosa* are often encoded on plasmids, such as IncP type; class I integrons, for example, those carrying the *bla*_VIM_ gene; and other MGE, such as those associated with insertion sequence common region (*IS*CR) elements [114]. In addition, these isolates frequently carry additional resistance determinants to fluoroquinolones and aminoglycosides. Carbapenemase-producing *P. aeruginosa* (CP-PA) is often resistant to these therapeutic options, thus making treatment failure likely. CP-PA has also been associated with nosocomial spread, prompting infection prevention interventions [280].

The Enterobacterales order, as defined by Adelou et al. in 2016, comprises Gram-negative, non-spore-forming, rod-shaped, and facultative anaerobes bacteria. The order contains the families *Enterobacteriaceae*, *Erwiniaceae*, *Pectobacteriaceae*, *Yersiniaceae*, *Hafniaceae*, *Morganellaceae*, and *Budviciaceae*, some of which are members of the normal microbiota of the mammalian gastrointestinal tract [282]. The drastic rise in the incidence of MDR and extended drug-resistant (XDR) pathogens belonging to the *Enterobacteriaceae* group is a significant economic problem as these pathogens are prevalent natural residents of the human and animal microbiomes and spread quickly between humans. Moreover, Enterobacterales easily acquire ARGs via MGEs [283].

Most notable in Enterobacterales is the resistance to β-lactam antibiotics due to ESBL production, mainly in *E. coli* and *K. pneumoniae*, followed by aminoglycoside and fluoroquinolone resistance (Table 1 and Table 2). These resistance phenotypes are often coupled, leading to MDR and the necessity to use last-resort antibiotics [284].

*K. pneumoniae* is the causative agent of about one-third of all Gram-negative infections (urinary tract infections, cystitis, pneumonia, surgical wound infections, endocarditis, septicemia, necrotizing pneumonia, pyogenic liver abscesses, and endogenous endophthalmitis), associated with high mortality rates and extended hospitalization, coupled with high economic costs. Due to selective pressure caused by antibiotic usage, *K. pneumoniae* collects ARGs, which led to the development of XDR strains harboring a ‘super resistome’. These include the emergence of hypervirulent *K. pneumoniae* (hvKp) or hypermucoviscous *K. pneumoniae* (HMKP), usually susceptible to last-line antibiotics (carbapenems and colistin) [285,286]. The continuous global dissemination of high-risk MDR and XDR *K. pneumoniae* highlights their complex evolution, involving the transfer and spread of ARGs and epidemic plasmids [287,288]. Most of the carbapenemase and/or ESBL-producing *K. pneumoniae* strains, as well as those harboring aminoglycoside resistance, belong to specific clones CC (clonal complex) 258, CC15, and CC14 [289], while colistin-resistant clones mainly belong to CC11, 43, and 258 [290,291,292,293].

*Enterobacter* spp. are increasingly described as contributing to the dissemination of infections caused by carbapenem resistant strains. Amongst the 22 species of this genus, *Enterobacter aerogenes, E. cloacae,* and *E. hormaechei* are the most frequently isolated species in clinical infections, mainly in immunocompromised patients and those hospitalized in ICU, due to their adaptation to the hospital environment and their ability to efficiently acquire numerous genetic mobile elements containing resistance and virulence genes [294].

*Enterobacter cloacae* complex (*E. cloacae*, *E. asburiae*, *E. hormaechei*, *E. kobei*, *E. ludwigii*, *E. mori*, and *E. nimipressuralis*) are common nosocomial pathogens involved in a wide variety of infections (pneumonia, UTI, and septicemia). The emergence of MDR clones, including resistance to the last-resort carbapenems, increased interest in these pathogens [295].

*E. coli* is particularly interesting since it represents a significant part of the normal microbiota, but it can also cause severe infections in humans and animals. In humans, *E. coli* can cause infections in practically every anatomical site of the human body, involving urinary tract infections, appendicitis, pneumonia, the bloodstream, gastrointestinal infections, skin abscesses, intra-amniotic and puerperal infections in pregnant women, meningitis, and endocarditis. Moreover, *E. coli* is involved in community-acquired and healthcare-related infections and can cause disease in all age groups [296,297].

*E. coli* is the second bacteria (after *Klebsiella*) involved in human infections associated with MDR bacterial infections. Furthermore, the significant increase in the emergence and dissemination of *E. coli* to the main antibiotic classes (β-lactams, quinolones, aminoglycosides, sulfonamides, and fosfomycin), including the last-resort carbapenems and polymyxins, is correlated with prolonged hospital stays and patient deaths [297,298].

### Other Enterobacterales

*Citrobacter* spp., mainly *C. freundii,* are inhabitants of the intestinal tract and have been associated with nosocomial infections involving the urinary tract, liver, biliary tract, peritoneum, intestines, bone, respiratory tract, endocardium, wounds, soft tissue, meninges, and the bloodstream. The emergence of MDR *Citrobacter* strains is an increasing concern due to the production of AmpC, broad-spectrum β-lactamase, ESBL, or even carbapenemase, particularly MBL or KPC types. In addition, quinolone resistance (*qnr* and *aac(6′)-Ib-cr* genes), numerous *qnrB* alleles, and about 40 *qnrB* variants (located on the chromosome of *Citrobacter* spp., especially *C. freundii*) were described [299].

*Hafnia alvei* is rarely isolated from human samples. When it does, it is responsible for nosocomial infections, including gastroenteritis, urinary tract infections, meningitis, pneumonia, wound infections, soft tissue infections, endophthalmitis, and septicemia. The organism resides in the gastrointestinal tract of humans and many animals. Most infections with *H. alvei* are identified in patients with severe underlying diseases (e.g., malignancies) or after surgery or trauma. Besides its natural resistance to colistin and expression of AmpC chromosomal β-lactamase, it was described as the emergence of a *Hafnia paralvei* resistant to carbapenems due to a defective porin [300].

*Morganella morganii* is ubiquitous and is often associated with stool specimens collected from patients with symptoms of diarrhea. They are normal inhabitants of the gastrointestinal tract. *M. morganii* has intrinsic resistance to oxacillin, ampicillin, amoxicillin, and most first- and second-generation cephalosporins, macrolides, lincosamides, glycopeptides, fosfomycin, fusidic acid, and colistin. AR in *M. morganii* has been raised in recent years, mainly due to MGEs, leading to MDR and XDR strains [301].

*Providencia* spp. are usually isolated from patients with urinary tract infections and diarrhea and are associated with nosocomial outbreaks. Most commonly, *P. rettgeri* and *P. stuartii* represent the majority of MDR strains isolated and are intrinsically resistant to penicillins and the first-generation cephalosporins, aminoglycosides, tetracyclines (including tigecycline), and colistin [302,303].

*Serratia* spp., most commonly *S. marcescens*, is involved in nosocomial outbreaks and the colonization of diverse healthcare settings. *S. marcescens* has been associated with meningitis, sepsis, UTIs, skin infections, bloodstream infections, and respiratory infections. The intrinsic resistance to ampicillin, first- and second-generation cephalosporins, macrolides, and antimicrobial peptides, including colistin, is very challenging for clinical management. Moreover, some strains express the SME-1 enzyme, conferring resistance to imipenem, aztreonam, cephalosporins, and penicillins [304,305].

*Salmonellae* are Gram-negative bacteria that are pathogenic to humans and are traditionally subdivided into two major groups based on their clinical presentation: typhoidal *Salmonella* and non-typhoidal *Salmonella*. Typhoidal *Salmonella,* comprising the *S. enterica* subspecies *enterica* (hereafter *Salmonella*) serovars Typhi and Paratyphi A, B, and C, cause a systemic disease also known as enteric fever [306]. Human-restricted *S.* Typhi is the dominant cause of typhoid fever, with an estimated number of cases between 21.7 million and 26.9 million per year [307] and an estimated 217,000 deaths per year [308]. *S. enterica* constitutes a significant public health concern, and it is estimated to cause more than 300,000 annual deaths, mostly in developing countries [309]. This species is classified into hundreds of serovars based on surface antigenic composition. Some serovars (e.g., *S.* Typhi and *S.* Paratyphi) are host-adapted to humans, where they cause a systemic infection known as typhoid or paratyphoid fever and are therefore referred to as “typhoidal” serovars. Other serovars, such as *S.* Typhimurium, have a broad host range and, in humans, most often cause self-limiting gastroenteritis and are referred to as “non-typhoidal” serovars [310].

In *Salmonella* spp., particularly *S.* Typhi, antimicrobial resistance could be mediated by plasmid or chromosomal DNA. Usually, resistance is developed by the inactivation of antibacterial agents, alteration of drug targets, and employing various efflux pumps. In addition, external resistance factors may be actively mediated by gene transfer using virulence plasmids, phages, and MGEs [311]. *S.* Typhi typically has plasmids that contain several virulence factors and ARGs. These plasmids vary in size (50–90 kb) and carry the *spv* operon, which is significantly involved in causing infection. The genes of this operon are reportedly pivotal for bacterial proliferation in host cells and supposedly enhance the virulence of the pathogen [312]. Considering that most virulence plasmids are not self-transferable, some contain *transgenes* that enable the transfer of plasmids via conjugation. Incompatible (Inc) plasmids encode multiple antimicrobial resistance genes in *S. Typhi* and are classified into IncH1, IncH2, and IncH3. In addition, plasmids R27, pHCM1, and pAKU1 comprise a composite transposon that can harbor multidrug resistance in MDR *S. Typhi* strains [313].

Regarding the production of β-lactamases, TEM, SHV, and CTX-M are the main types of ESBLs in *Salmonella* spp., conferring resistance to penicillin and cephalosporin [314]. In *S.* Typhi, the presence of these genes has been attributed to the genetic transfer of resistance genes from other Gram-negative bacterial species [315]. In *Salmonella* spp., there were described genes encoding resistance to tetracycline (*tetA, tetB, tetG*), quinolones (*qnrA, qnrB, qnrC, qnrS),* and chloramphenicol (*cat1* and *cat2*). Genetic elements identifying the mobile gene cassettes that carry multidrug-resistant genes are known as integrons. In *S.* Typhi, the presence of integrons (classes 1 and 2) equalizes the distribution of antimicrobial resistance, in which class 1 is more dominant [316].

## 4. Antibiotic Resistance in Romania

### 4.1. Antibiotic Resistance in Romanian Hospital Settings

Romania is one of the European countries with the highest rates of MDR in *A. baumannii* clinical isolates (in 2020, the highest resistance percentages were recorded for fluoroquinolones, carbapenems, and aminoglycosides) [317]. Concerning *P. aeruginosa*, the highest resistance levels in 2020 were recorded for fluoroquinolones, carbapenems, and ceftazidime [318]. To the best of our knowledge, available data on molecular characterization of the non-fermenting strains is sourced from the major geographical areas of the country, including the capital city (Table 3).

Clinical *A. baumannii* strains from different Romanian regions exhibited CHLD-producing *bla*_OXA-23_ (West, North, Central, and South regions), followed by *bla*_OXA-24/72_ gene (North, Central, and South regions), and *bla*_OXA-58_ gene revealed by one study from the capital city, and respectively MBLs encoding *bla*_VIM-2_ and *bla*_IMP-1_ genes (South and North). The distribution of CHLDs and MBLs by isolation sources and period highlighted that carbapenem-encoding genes were not correlated over the period with the specific isolation sources, being described in strains isolated from infection sites or anal carriage. Studies from the West, Central, and South regions reported ESBL-encoding genes (*bla*_PER-1_; *bla*_TEM-1_; *bla*_TEM-12_; *bla*_TEM-84_ and *bla*_SHV-12_) from sterile or non-sterile isolation sources (Table 3). However, a limited number of studies have focused on the MGE carrying the CHLDs and revealed the presence of class 1 integrons, insertion sequences, transposons, or different plasmid types in *A. baumannii* recovered from South and Western Romanian intrahospital infections. *A. baumannii* reported from western and southern Romania belonged to high-risk international clones like ST2, ST1, ST636, and ST492. 

Between 2008 and 2015, the most common carbapenemase-encoding genes in *P. aeruginosa* clinical strains isolated from intra-hospital infections or carriages in North, Central, and South Romania were *bla*_VIM-2_, *bla*_VIM-4_ and *bla*_IMP-13_. In addition, ESBL-encoding genes (*bla*_SHVlike_; *bla*_GESlike_; *bla*_VEBlike_; *bla*_TEMlike_) were encountered in *P. aeruginosa* strains isolated in the southern region of Romania.

Clinical enterobacterial isolates harbored mainly the ESBL *bla*_CTX-M(-15)_ gene, followed by *bla*_SHV_ and *bla*_TEM_ (for which the ESBL phenotype depends on the gene variant). *bla*_OXA-48_ was the most commonly reported carbapenemase, followed by *bla*_NDM_ and *bla*_KPC_ (Table 4). Aminoglycoside resistance was mainly associated with AMEs, and all variants of PMQR have been described (Table 5).

Several studies investigated the mobile genetic platforms carrying the respective ARGs, particularly in the southern part of the country, and highlighted the presence of plasmids and integrons previously associated with AR without being associated with specific isolation sources. In addition, strains isolated from different infection sites of inpatients from hospitals in the south of Romania belonged to widespread international *E. coli* (e.g., ST131, ST10, and ST5) or *K. pneumoniae* (ST101) clones.

In 2020, the reported antibiotic resistance levels in Enterobacterales indicated that *K. pneumoniae* had the highest resistance levels in third generation cephalosporins, fluoroquinolones, aminoglycosides, and *E. coli* for aminopenicillins, fluoroquinolones, and third generation cephalosporins [317].

Several encoding genes and MGE (class 1 integrons and Inc plasmids) were reported in different parts of Romania (Table 4 and Table 5).

### 4.2. Community-Acquired Antibiotic Resistance

Generally, infections are classified into two categories: community-acquired and nosocomial (intra-hospital) infections. Healthcare-associated infections are specific to admitted hospital patients and occur after at least 48 h from admission, while community-acquired infections are contracted outside of a healthcare facility and diagnosed within 48 h after admission (community onset) [346,358].

The occurrence of AR in community-acquired infections is increasing due to multiple factors. Antibiotic overuse, for example, can imbalance the composition of the gut microbiota, facilitating the emergence and colonization of the gut with antibiotic-resistant bacteria (ARB) and the proliferation of opportunistic pathogens [359]. On the other hand, antibiotic residues in the environment or in food products could select for resistance [360,361].

Community-acquired AR was scarcely investigated in Romania (Table 6). For *E. coli*, we have identified five studies reporting carbapenemases, ESBLs, and aminoglycosides, PMQR, trimethoprim, and tetracycline resistance genes in strains isolated from community-acquired UTIs. In *A. baumannii*, the presence of CHLDs, AMEs, and sulphonamide, tetracycline, and macrolide resistance genes has beeen reported, while in *P. aeruginosa*, the presence of MBL, AMEs, and PMQR, sulphonamide, trimethoprim, and tetracycline resistance genes has been reported in strains isolated from all geographical regions of the country. Furthermore, class 1 and 2 integrons were involved in disseminating ARGs in these strains.

### 4.3. Antibiotic Resistance in Veterinary Settings

Several factors can affect the occurrence and dissemination of AR in the animal industry, including antibiotic use and farm management. Many studies have focused on how the use of antibiotics in food-producing animals has led to the expansion of antibiotic resistance. In industrialized countries, the companion animal population has dramatically increased during the last few decades. The increased interaction between animals and humans leads to a higher risk of infections and the cross-transmission of AR traits. Thus, the potential of reverse zoonosis and the creation of animal reservoirs that keep the loop of infection and AR diffusion open are gaining steadily increasing concern. Antimicrobial resistance of pet origin, responsible for both direct and/or indirect threats to human health, involves mainly carbapenemase-producing enterobacteria and ESBL Gram-negative bacteria [366].

The epidemiological scale of AR transmission between humans and animals is not yet well defined, as multiple parameters should be taken into account (population features, geographical location, investigative methods), and the sole abuse or misuse of antibiotics is insufficient for such a massive transmission of resistant microorganisms between humans and pets [367,368]. Therefore, several research lines are being explored, such as human-animal transmission and vice versa, although controversial results are being observed [369]. Moreover, the environment most likely contributes to AR dissemination, intended as the vector connecting the human and animal environments, including anthropic activities. It was also suggested that monitoring non-pathogenic specimens and their potential capability to acquire resistance traits is a promising strategy to predict and prevent future resistant strains.

In Romania, very scarce information is available regarding the isolation, identification, and AR of Gram-negative bacilli from veterinary settings; the ARGs and carrying platforms were investigated only for Enterobacterales species, reported in three studies (Table 7). The other studies reveal only the presence of resistant bacterial strains in different animal isolates without investigating the genetic background of AR.

Thus, gentamycin- and penicillin-resistant *Pseudomonadaceae* strains were described in samples from boar semen from three artificial insemination centers in the northwest of Romania [367]. Cristina et al. investigated the presence of AR in isolates from pet reptiles (chelonians, snakes, and lizards) and identified the presence of *P. aeruginosa*, *Citrobacter koseri*, *C. brakii,* and *K. oxytoca* resistant to cephalosporins (up to the fourth generation), tetracyclines, quinolones, aminoglycosides, and others [370]. Tîrziu et al. investigated the prevalence and AR profiles of two major foodborne pathogens (*Salmonella* spp. and, respectively, *Campylobacter* spp.) in different food products from two Transylvanian counties of Romania and revealed high levels of resistance to tetracycline, ciprofloxacin, and nalidixic acid in both pathogens [371]. A high level of AR in *Campylobacter* spp. was also reported in strains isolated from broiler chicken flocks from three north-western Transylvanian counties of Romania [372].

**Table 7:** Genetic background of AR and carrying platforms in Gram-negative strains isolated in Romanian veterinary settings.

Species	Location/Year	Isolation Source	β-Lactam Resistance Genes	Aminoglycoside Resistance Genes	Quinolone Resistance Genes	Other ARGs	MGEs	Reference
*E. coli*, *K. pneumoniae*	North-East and South-East Romania 2017–2018	dog faecal samples	bla_CTX-M-1, -3, -9, -14, -15_; * bla_T_*_EM-1;_ * blas*_HV-2, -52;_	*aph(3′)-Ia*; *aph(6)-Id*; *ant(3″)-Ib*; * aac(3)-IId*; * aac(2′)-IIa*;	*qnrS1*	*mph*; * sul1*, *-2*; * tet(A)*, *-(B)*	Inc plasmids: HI2; Y; P1; FIB; F; N; F2A; L/M FIB/K; I1 FIB/Y/FIA;	[355]
*E. coli*	Timiș and Arad counties 2019–2020	swine intestinal microbiota	-	-	*qnrB*, *-S*	-	-	[373]
*E. coli* *Salmonella* spp.	Cluj-Napoca 2012–2013	chicken carcasses	*bla_TEM_*	*aadA1*	*-*	*tet(A)*, *sul1*, *dfrIa*	-	[374]
*E. coli*	Romania, 1980–2016	rectal swabbing of calves, foals, and piglet	*bla_TEM_*	*-*	*-*	*tet(A)*, *tet(B)*, *tet(C)*, *sul1*, *dfrA1*	*int1*, *int2*	[375]

### 4.4. Antibiotic Resistance in Gram-Negative ESCAPE Pathogens in Wastewaters

Antibiotics are among the most popular pharmaceuticals used in human medicine, veterinary care, and farming [376,377,378]. They are also frequent contaminants in wastewater, municipal sewage, and wastewater treatment plants’ influents and effluents [379]. Hospitals generate an impressive amount of wastewater per day; the hospital effluents are loaded with pathogenic microorganisms, antibiotics, and other pharmaceutical or toxic substances, which are only partially removed during wastewater treatments, contributing to the pollution of the natural environment, including the selection and dissemination of AR [380,381]. Wastewater treatment plants (WWTPs) are one of the critical reservoirs of both antibiotic-resistant bacteria and ARGs and represent hotspots for horizontal gene transfer (HGT) via MGEs, such as plasmid integrons, transposons, resistance islands, and insertion sequences, enabling the development and dissemination of ARGs between bacteria [382,383]. Antibiotic-resistant bacteria are collected and mixed with environmental strains, which, in turn, could introduce the newly acquired ARGs into the clinics [384]. Romania had the third-highest consumption of antibacterials for systemic use in the community sector in 2019 [385]. In 2016 and 2018, the most-consumed classes for food-producing animals in Romania, according to the European Surveillance of Veterinary Antimicrobial Consumption (EVSAC), were tetracyclines and penicillins, respectively [386]. This explains the presence of antibiotics in WWTP, representing a high selective pressure for AR [387]. In this context, during the last few years, international authorities have made considerable efforts to improve the monitoring of the circulation of the antibiotic-resistant bacteria in different environments, underscoring the necessity to strengthen intersectional human, animal, and agricultural cooperation, which has been included as a priority in the work plan for the EU Health Programme. One of the priority topics of the Joint Programme Initiative on Antimicrobial Resistance (JPIAMR) is the elucidation of the role of the environment as a source for the selection and dissemination of AR, which is expected to provide essential data for monitoring AR, as the lack of surveillance is considered one of the main contributors to the spread of AR, particularly in developing countries. In this regard, one important goal is mapping the distribution of MDR strains and plasmids and different genomic lineages of critical nosocomial pathogens in different clinical and aquatic compartments. This vital knowledge could be translated into policy measures to control the emergence and spread of antibiotic-resistant bacteria [388,389].

Contrary to clinical studies, there needs to be more information regarding the ARG reservoirs in the wastewater network in Romania. Our research team showed a high repertoire of ARGs and virulence markers in *K. pneumoniae* ST101 isolated from intra-hospital infections and wastewater samples collected from the influent and, respectively, from the effluent of hospital collecting sewage tanks in the southern regions of Romania; the transmission of MDR, carbapenemase and ESBL-producing *K. pneumoniae* ST101 from hospital to hospital effluent; and its persistence after the chlorine treatment [339,344]. In the country’s central region, a chlorinated wastewater treatment system from a public hospital revealed the presence of the following carbapenemase and ESBL-encoding genes in the influent: *bla*_PER_, *bla*_VIM_, *bla*_NDM-1_, and *bla*_SHV_. In contrast, the chlorinated effluent exhibited *bla*_VIM_ and *bla*_SHV_ [390]. Another study performed in Cluj-Napoca on one WWTP and the receiver river Someșul Mic revealed the presence of tetracycline and sulphonamide ARGs [*sul1*, *tet(O)*, and *tet(W)*] in wastewater without focusing on total antibiotic-resistant bacteria or ARGs identification [391]. Butiuc-Keul et al., in 2019, revealed the genetic background of AR in *Pseudomonas* spp. from urban water sources and their environmental impact in north-western Romania [392]. Several carbapenemase-encoding genes have recently shown spatiotemporal variation in wastewater samples from the influent and effluent of three Cluj-Napoca WWTPs [393]. In South Romania, Van et al. recently revealed the efficiency of commercial essential oils against antibiotic-resistant *P. aeruginosa* clinical and wastewater strains [394]. Gheorghe-Barbu et al. have demonstrated a high repertoire of ARGs in *A. baumannii* and *P. aeruginosa* strains isolated two years consecutively from intra-hospital infections, wastewater, and surface water from three geographical regions of Romania and highlighted the importance of screening for acquired antimicrobial resistance in the environment [330] (Table 8).

### 4.5. Antibiotic Resistance in Other Aquatic Ecosystems

Surface water plays an essential role in AR dissemination by being both a habitat and a dissemination ecosystem for microorganisms. Recently, Banciu et al. demonstrated the dissemination of *A. baumannii* and *P. aeruginosa* clinical strains in wastewater or surface water or the presence of *E. coli*, *K. oxytoca, C. freundii*, and *P. mirabilis* resistant to ampicillin and clavulanic acid, strains isolated from the St. Gheorghe branch of the Danube Delta [386]. The Danube River is considered the most critical non-oceanic body of water in Europe and the “future central axis for the European Union,” Its Danube Delta is included in the Biosphere Reserve and Ramsar Site lists. The Danube River crosses ten countries. This basin represents an optimal pool for resistant pathogens and anthropogenic pollutants dissemination and accumulation throughout large and distant areas, being assigned as a reservoir of AR. Previously, it has been demonstrated that Bucharest was at the top of the most polluted sampling locations from twelve WWTPs in nine countries (Romania, Serbia, Hungary, Slovenia, Croatia, Slovakia, Czechia, Austria, and Germany) in the Danube River Basin collected and analyzed for ARGs and MGE presence [395].

Several other authors characterized at the molecular level the Gram-negative rods from the surface water (Dambovița river—south Romania), from the 4 Romanian natural aquatic fishery lowland salted lakes from the Natura 2000 Network located in Buzǎu and Brǎila counties, carrying a high diversity of resistance markers correlated with class one integrons [396] (see Table 8).

**Table 8:** Genetic background of antibiotic resistance and carrying platforms in Gram-negative strains isolated in Romania from wastewater and surface waters.

Species	Location/Period	Isolation Source	β-lactam Antibiotics Genes	Aminoglycoside Resistance Genes	Quinolone Resistance Genes	Other ARGs	MGEs	Reference
*A. baumannii*	Bucharest, Târgoviște, Vâlcea, Iași, Galați, Timișoara, Cluj; 2018–2019	hospital sewage tanks, WWTPs—influent, active sludge, effluent; surface water—upstream and downstream region (200 m) of the rivers: Dambovita, Ialomita, Olt, Bahlui, Siret, Bega, Somes	*bla*_TEM-1, -12 _ *bla*_OXA-23_; *bla*_OXA-72_; *bla*_OXA-65, -66, -126, -217_; *bla*_ADC-5, -25, -73, -81, -154, -167_;	*aph(6)-Id*; * aph (3′)-VIa*; * ant(3″)-Ib*, *-IIa*; * aac(3)I*, *-Ip*; * aadA1*, *-A2*; * armA*; * ant(2″)-Ia*;	-	*catA1*; *catB8*; *cmlB1*; *sul1*, *-2*; *tet(A)*, *-(B)*; *mph(E)*; *msrE*; *drfA12*;	*qacE*∆*1* integron associated gene	[330]
*P. aeruginosa*	Bucharest; 2018–2019; Târgoviște, Vâlcea, Iași, Galați, Timișoara, Cluj; 2018–2019	-	*bla_GES-4_*; *bla_VEB-9_* *bla*_TEM-40_; *bla*_IMP-13_; *bla*_VIM-2_;	*aac(6′)-II*; *aadA1*, *-A2*; *aph(3′)-Ia*, *-IIb*; * aac(6)-Id*, *-II*; * ant(2″)-Ia*	-	*fosA*; * catB7*, *bcr1*; * tet(A)*, *-(G)*; *mphE*; *msrE*;	*qacE*∆*1* integron associated gene	[330]
*P. aeruginosa*	Bucharest 2018–2020	intra-hospital infections, hospital sewage tank, WWTPs	*bla*_CTX-M_; *bla*_SHV_; *bla*_GES_; *bla*_VEB_; *bla*_VIM_	*-*	-	*-*	-	[393]
*Pseudomonas* spp.	Cluj-Napoca 2015	hospital effluent, municipal WWTP, Somesul Mic river—upstream and downstream	*bla*_TEM-1_; *bla*_SHV-1_; *bla*_PER-1_; *bla*_VIM-1_; * bla*_PstS_	*aac(6′)-II*; *aac(3)-IIIa*	*qnrA*, *-B *	*ermB*; * tet(A)*, *-(B)*, *-(C) *	*intI*	[391]
not provided	Cluj county, 2019–2020	influent, effluent samples from 3 WWTPs	*bla*_KPC_; *bla*_IMP;_ *bla*_NDM_; *bla*_OXA-48_	*-*	-	*-*	-	[390]
*S. marcescens*, * K. pneumoniae*, *K. oxytoca*, *E. coli*, *E. cloacae* complex, *A. calcoaceticus*, *R. orniyhinolytica*, *E. hermanii*, *E. cowanii*, *S. rubidaea*, *P. ananatis*, *H. alvei*	Buzău, Brăila counties 2016	Four lowland salted lakes included in Natura 2000 network	*bla_CTX-M_ (S. marcescens*, *K. oxytoca*, *A. calcoaceticus*, *E. cloacae*, *E. coli*, *H. alvei) * bla_NDM_ (*E. coli*) *bla_IMP_* (*E. kobei*)	*aac-(3)Ia (K. pneumoniae)*	*qnrS (E. cloacae)*	*sul1*	*intI*	[397]
*K. pneumoniae*	Bucharest 2018–2019	hospital collecting sewage tank (influent, effluent)	*bla*_CTX–M–15_; * bla*_OXA–1_; * bla*_OXA–48_; * bla*_SHV-1, -12, -106, –107, -145, -158, -187_; * bla*_KPC-2_; * bla*_TEM–1, -150_; *(K. pneumoniae)*	*aac(3)II-a*; *aph(6)-Id*; *ant(3″)-Ib*; *aadA2 (K. pneumoniae)*	*qnrS1*; *qnrB1*; *qnrB4*	*tet(A)*, *-(D)* *catA1*, *-A2*; *sul1*, *-2*; * arr2*, *-3*; * dfrA1*, *-A12*, *-A14*; * msrE*; *mphE*; *cmlA5 (K. pneumoniae)*;	qacE∆1	[344]
*K. pneumoniae*	Bucharest, Galați and Târgoviște 2018–2019	wastewater—influent, effluent from WWTPs	*bla*_CMY-4_; *bla*_CTX-M-15_ *bla*_DHA-1_; *bla*_KPC-2_; *bla*_NDM-1_ *bla*_OXA-1, -9, -10_; *bla*_OXA-48, -162_; *bla*_SHV-1, -11, -12; -100, -107, -145, -158, -161_; *bla*_TEM-1, -150_;	*aac(3)-IIa*, *-IId* *aac(6′)-Ib*, *-Ibcr*, *-IId*, *-Il*; * aadA1*, *-A2*, *-A5* *ant(2″)Ia* *aph(3′)-Ia*, *-Ib*; * aph(6)Id*;	*qnrB1*, *-B4-B10*, *-B19*, *-B36*, *-B67* *qnrD1* *qnrS1*	*tet(A)*, *-(D)* *catA1*, *-A2*; * catB3*; *cmlA5*; *fosA*, *-A6*; * mphA*;*mphE*; *msrE*; * arr2*, *-3*; * dfrA1*, *-A12*, *-A14*; * sul1*, *-2* *armA*;*ble*;	*qacEdelta1*	[339]
Gram-negative rods	Bucharest, 2011–2012	hospital sewage; influent, effluent of WWTPs; surface water—Dâmbovița river—upstream, downstream after the WWTP discharge	*bla*_TEM_, *bla*_SHV_, *bla*_CMY_, *bla*_CTX-M_, * bla*_NDM_, *bla*_VIM_	*-*	*qnrB*; *qnrS*	*tet(B)*, *-(M)* *sulII*; *dfrA1-aadA1*;	-	[398]
not provided	Cluj 2017	influent, effluent of WWTPs; surface water—Someșul Mic river- upstream, downstream after the WWTP discharge	*-*	*-*	-	*sul1*; *tet(O)*, *-(W)*;	intI1	[391]
not provided	Cluj county 2015	hospital sewage tank (influent, effluent)	*bla*_PER_; *bla*_VIM_; *bla*_NDM-1_; *bla*_SHV_;	*-*	-	*-*	-	[390]
*Acinetobacter* spp., *Enterobacteriaceae*, *Pseudomonas* spp.	Cluj county 2015	hospital sewage tanks	*bla_VIM_*; *bla_SHV_*;	*aacC2*	-	*sul 1*, *-2*; *qacE*, *tet(A)*, *-(B)*, *-(C)*, *-(W)*; *catA1*; *floR*;	-	[399]
*Enterobacter spp.*, *E. coli*, *K. pneumoniae*	Danube river 2013	surface water	*bla*_CTX-M-15, -3, -9, -27, -55_; *bla*_SHV-1,-2,-11,-12_; *bla*_KPC-2_; *bla*_NDM-1_,	*-*	-	*-*	-	[400]
not provided	Bucharest 2017	wastewater (WWTP)	*bla*_OXA_; *bla*_SHV_	*aph(III)a*	*qnrS*	*tet(B)*; *tet(M)*; *sul1*; *ermB*; *ermF*; *vanA*	intI1	[396]
not provided	Cluj 2017	wastewater (WWTP)	*bla*_OXA_; *bla*_SHV_	*aph(III)a*	*qnrS*	*tet(M)*; *sul1*; *ermB*; *ermF*	intI1	[396]

## 5. Conclusions

Updated information regarding the genetic background and molecular epidemiology of AR is crucial for tackling the spread of this phenomenon. This review brings together the available data regarding the AR of Gram-negative ESCAPE pathogens circulating in Romania. The big picture for Gram-negative ESCAPE pathogens reveals that all significant, clinically relevant, globally spread ARGs and carrying platforms are well established in different areas of our country and are already disseminated beyond clinical settings.

To constrain the spread of ESCAPE pathogens, it is now well recognized that collaborative efforts are required by policymakers, funders, and those responsible for the treatment and management of ESCAPE pathogens. Aside from novel drug development, these collaborative endeavors will require sustainable stewardship practices to reduce the inappropriate use of antibiotics in both the human health and agricultural sectors. In addition, improvements in factors encompassing AR surveillance, diagnostics, patient education, and patient treatment options will help facilitate AR control.

## Figures and Tables

**Table 3:** Transferable β-lactamase encoding genes described in *A. baumannii* and *P. aeruginosa* nosocomial strains isolated in Romania.

Species/Clone	City and Year of Isolation	Isolation Source	ARGs	MGEs	Reference
*A. baumannii*	Bucharest 2001–2003	not provided	*bla*_IPM-1_, *bla*_VIM-2_, *bla*_OXA-24_, *bla*_OXA-51,_ *bla*_OXA58_	class 1 integrons	[319]
*A. baumannii*	Iași 2003–2007	not provided	*bla*_VIM-2_	-	[320]
*A. baumannii*	Iași 2008–2012	urine, pus, sputum, tracheal aspirate, blood, cerebrospinal fluid	*bla*_VIM-2_	-	[316]
*A. baumannii*	Petroșani 2004	rectal swabs	*bla*_PER-1_ *bla*_TEM-1_	Tn*1213*	[321]
*A. baumannii* ST1; ST2	Timișoara, Arad and Reșita 2009–2010	bronchial aspirates, wound secretions, catheters, stool, blood, urine cultures, tracheal secretions	*bla*_OXA-23_ *bla*_PER-1_ *bla*_TEM-1_	IS*Aba1*	[322]
*A. baumannii*	Iași and Tg.-Mureș 2014–2015	stool, blood, urine, tracheal secretions, stool samples	*bla*_OXA-23_, *bla*_OXA-24/72_ * bla*_SHV-12_	-	[323]
*A. baumannii* ST437, ST764, ST765	Bucharest 2011–2012	tracheal secretions, wounds, blood, urine, catheters, stool	*bla*_OXA-23_	aci6 (pABKp1-like)	[324]
*A. baumannii*	Bucharest 2012–2013	not provided	*bla*_SHV-like_	-	[325]
*A. baumannii*	Bucharest 2011–2012	wound secretions, nasal–pharyngeal exudates, sputum, bronchoalveolar lavage, stool, urine, blood, pleural fluid, catheters	*bla*_OXA-23_	-	[326]
*A. baumannii*	Bucharest 2014–2015	catheter, tracheal secretion, nasal exudate	*bla*_OXA-23_, *bla*_TEM_	-	[327]
*A. baumannii*	Bucharest 2015	chronic leg ulcer samples	*bla*_OXA-72_	-	[328]
*A. baumannii*	Bucharest 2017	not provided	*bla*_OXA-23_,	-	[329]
*A. baumannii ST231*	Bucharest 2015	wound secretions left ear	*bla_OXA-23_*, *bla_OXA-64_*	-	[330]
*A. baumannii*	Bucharest 2015–2016	nasal secretions, tracheal secretions	*bla_OXA-23_*	-	[331]
*A. baumannii ST502*	Romania 2015–2017	lower respiratory tract, blood, skin or soft tissue, urine, intra-abdominal fluid, wound	*bla_OXA-24_*, *bla_OXA-23_*	-	[332]
*A. baumannii*	Bucharest 2017	not provided	*bla*_IPM_, *bla*_VIM-2_, *bla*_OXA-23_, *bla*_OXA-51_	Aci1, pACICU2, rep135040, p3S18, Aci6	[333]
*A. baumannii ST636*, *ST492*, *ST1*, *ST2*, *ST642*, *ST312*	Bucharest 2017–2018	blood, urine, pharyngeal exudate, stool, anal carriage, tracheal secretion, catheter, cerebrospinal fluid, sputum	*bla*_OXA-23_, *bla*_OXA-24,_ * bla*_TEM-84_ *bla*_TEM-12_ *bla*_PER-1_	class 1 integrons (5′-CS—dfrA12—aadA2—3′-CS; 5′-CS—aac(3)-I—aadA1—3′CS); plasmids (pACICU2-like; pMAL-1 like—*bla*_OXA-72_	[29]
*A. baumannii*	Bucharest, Târgoviște, Vâlcea, Iași, Galați, Timișoara and Cluj 2018–2019	intra-hospital infections; not provided sources	*bla*_OXA-72_; * bla*_OXA-23_; *bla*_OXA-66_; * bla*_OXA-65;_ * bla*_TEM-12;_ * bla*_ADC-30_	qacE∆1 integron associated gene	[334]
*P. aeruginosa ST233*, *ST364*, *ST1074*	Bucharest 2012–2013	tracheal secretions, wounds, blood, urine, catheter, stool samples	*bla_VIM-2_*	-	[324]
*P. aeruginosa*	Cluj Napoca 2010	respiratory, urinary tract, and postoperative wound infections	*bla_OXA-50_*, *bla_OXA-2_*	-	[335]
*P. aeruginosa*	Iași 2008–2012	urine, pus, sputum, tracheal aspirate, blood, cerebrospinal fluid, catheter	*bla_VIM-2_*	class 1 integrons (*IntI1-aacA7-bla_VIM-2_-qacE*∆*1* and *IntI1-aacA7-*∆*bla_VIM_-*∆*cmlA1-qacE*∆*1*)	[320]
*P. aeruginosa*	Iași and Tg.-Mureș 2014–2015	stool samples	*bla*_VIM-2_	-	[323]
*P. aeruginosa*	Bucharest 2012–2013	not provided	*bla*_SHVlike_ * bla*_GESlike_ *bla*_VEBlike_	-	[325]
*P. aeruginosa*	Bucharest 2011–2012	wound secretions, nasal–pharyngeal exudates, sputum, bronchoalveolar lavage, stool, urine, blood, pleural fluid, and catheters	*bla_VIM-4_ bla*_SPM-like_ *bla*_GESlike_	class 1 integrons (*aacA7*- *bla*_VIM-4_ *aadB*)	[326]
*P. aeruginosa* ST2026, ST1982	Cluj-Napoca 2011–2013	blood, urine, ear	*bla*_VIM-2_, *bla*_IMP-13_	*IncFIC*	[336]
*P. aeruginosa*	Bucharest 2014	chronic leg ulcers	*bla*_IMP_	-	[337]
*P. aeruginosa*	Bucharest 2015	not provided	*bla*_IMP_	-	[338]
*P. aeruginosa*	Cluj-Napoca 2016	pus, tracheal secretion, bile, sputum, blood, central venous catheters, urine, nasal secretion, stool	*bla*_VIM_ *bla*_IMP_	-	[339]
*P. aeruginosa*	Bucharest 2014–2015	stool samples, tracheal secretion	*bla*_TEM_	-	[327]
*P. aeruginosa*	Bucharest 2014–2015	wound secretions, blood	*bla*_IMP_	-	[340]
*P. aeruginosa*	Bucharest 2016	hospital surfaces	*bla*_TEM_		[341]
*P. aeruginosa*	Bucharest, Târgoviște, Vâlcea, Iași, Galați, Timișoara and Cluj 2018–2019	intra-hospital infections (sources not provided)	*bla*_IMP-13_; * bla*_VIM-2_; * bla_VEB-9_*; * bla_TEM-40_*;		[334]

**Table 4:** Genetic background and carrying platforms of β-lactam resistance in Enterobacterales clinical strains isolated in Romania.

Species/Clone	City and Year of Isolation	Isolation Source	ESBL Genes	Carba- Penemases	MGEs	Reference
*E. coli*	Iași 2012	urinary catheter	*bla*_CTX-M-15_	-	-	[342]
*K. pneumoniae* ST525, *E. cloacae*, *E. coli* ST131, *K. pneumoniae* ST101, *Serratia marcescens*, *K. pneumoniae*, *S. marcescens*	Târgu Mureș 2013	blood, pus (not provided for all strains)	*bla*_CTX-M-15_	*bla*_NDM-1_, *bla*_OXA-48, -181_		[343]
*K. pneumoniae*, *E. cloacae*	Bucharest 2014	wound secretions, nasal–pharyngeal exudates, sputum, bronchoalveolar lavage, stool, urine, blood, pleural fluid, and catheters	-	*bla*_OXA-48_, *bla*_NDM-1_	-	[326]
*E. coli*	Bucharest 2014	not provided	-	*bla*_NDM-1_	-	[325]
*Enterobacteriaceae*	Bucharest 2014	not provided	*bla*_CTX-M_, *bla*_TEM_, *bla*_SHV_	-	-	[325]
*K. pneumoniae*	Bacău 2015	not provided	-	*bla*_VIM-1_	-	[344]
*K. pneumoniae*	Iași 2015	pharyngeal secretion, stool samples, feeding tub, bedside, food jar	*bla*_CTX-M-15,_ *bla*_CTX-M-55/79_	-	-	[323]
*K. pneumoniae* ST147, ST395; *E. cloacae* ST114, *P. stuartii*	Bucharest 2015	perineum, rectum	*bla*_CTX-M-15_, *bla*_OXA-1, -10_, * bla*_CMY-4_, *bla*_ACT-16_	*bla*_OXA-48_, *bla*_NDM-1_		[330]
*E. coli*, *Enterobacter*, *Proteus*	Cluj-Napoca 2016	urinary tract infections, wound infections, patients with persistent diarrhea	*bla*_CTX-M_		*intI1* (not related to *bla*_CTX-M_)	[345]
*K. pneumoniae*, *E. coli*	Bucharest 2016	hospital surfaces	*bla*_TEM_, *bla*_CTX-M_	*bla*_NDM_		[342]
*E. coli* ST5; *E. hormachei* ST74, ST171; * K. pneumoniae* ST101, ST395	Bucharest 2016	stool, bronchial secretion, pleural fluid, tracheal secretion, peritoneal fluid	*bla*_CTX-M_	*bla*_NDM_, *bla*_OXA-48, -181_	IncFIIγ, IncL, IncR	[346]
*K. pneumoniae*	Iași 2017	urine, lower respiratory tract, blood, wound, puncture, and peritoneum sources	-	*bla*_NDM-1_	-	[347]
*K. pneumoniae*	Bucharest 2017	urine, lower respiratory tract, blood, wound, puncture, and peritoneum sources	-	*bla*_NDM-1_, *bla*_KPC-2_, *bla*_VIM-1_	-	[344]
*K. pneumoniae*, *M. morgannii*, *E. tarda*	Bucharest 2017	urine	*bla*_CTX-M,_ *bla*_TEM,_ *bla*_SHV_	-	-	[348]
*E. coli*, *K. pneumoniae*, *E. asburiae*, *Citrobacter freundii*	Bucharest 2017	stool samples, blood	*bla*_TEM_, *bla*_CTX-M_	*bla*_OXA-48_	-	[349]
*K. pneumoniae*	Bucharest 2018	not provided	*bla*_CTX-M,_ *bla*_TEM_	*bla*_NDM_, *bla*_OXA-48_	-	[329]
*E. coli*	Craiova 2021	purulent secretion, tracheal aspirate, catheter	*bla*_CTXM-15_; *bla*_TEM-1_	-	-	[345]
*K. pneumoniae*	Craiova 2021	sputum, peritoneal fluid, purulent secretion, tracheal aspirate, catheter, wound secretion	*bla*_CTX-M-15_, *bla*_SHV-1_; *bla*_TEM-1_			[345]
*K. pneumoniae*	Bucharest 2021	urinary tract infections	*bla*_TEM_	*bla*_OXA-48_		[350]
*K. pneumoniae* ST101	Bucharest 2021	not provided	-	*bla*_NDM-1_		[351]
*E. coli*, *K. pneumoniae*, *P. mirabilis*, *Citrobacter sp*., *M. morganii*, *P. stuarti*, *E. cloacae*, *S. marcescens*, *P. rettgeri*	Bucharest 2022	surgical wound infections	*bla*_TEM_, *bla*_SHV_, *bla*_CTX-M_	*bla*_OXA-48_		[352]
*E. coli* ST131, ST10, ST131, ST167, ST410, ST540, ST1275, ST10, ST167	Bucharest 2010–2012	blood	*bla*_CTX-M-15_	-	*IncF*	[353]
*Enterobacterales* (*K. pneumoniae*, *E. cloacae*, *E. coli*; *P. mirabilis*; *S. marcescens*; *S. liquefaciens*; *S. plymuthica*)	Tg.-Mureș 2012–2013	respiratory tract infections, skin, and soft tissue infections, urine, blood, stool samples, catheter tips, bile, cerebrospinal fluid, pleural fluid, peritoneal fluid	-	*bla*_OXA-48_, *bla*_NDM-1_	IncR, L, FIIK, FII, FIB KN, HI2, M, A/C	[354]
*E. coli*	Iași 2017–2018	stool samples	*bla*_CTX-M-1, -3, -14, -15_ *bla*_TEM-1, -55_*bla*_SHV-134_ * bla*_SHV-like_;	-	Inc FIB/FIA; F; F/I1; I1; L/M; HI2; P1; N; Y; HI2	[355]
*Enterobacterales*	Bucharest 2017–2018 2017–2018	urine, bronchial secretions, blood, ascites fluids, abscesses, catheters		*bla*_KPC_; *bla*_OXA-48_; *bla*_NDM_		[356]
*K. pneumoniae* ST101, ST219	Bucharest, Galați and Târgoviște 2018–2019	not provided	*bla*_CTX-M-15_; * bla*_TEM-1, -150_; *bla*_SHV-11, -12, -33, -100, -101, -106, -107, -145, -158, -161, 187_	*bla*_NDM-1_; *bla*_KPC-2_ *bla*_OXA-48_	*qacE*∆*1*	[357]

**Table 5:** Genetic background and carrying platforms of non-β-lactam ARGs in Enterobacterales clinical strains isolated in Romania.

Species	City and Year of Isolation	Isolation Source	Aminoglycoside Resistance	Quinolone Resistance Genes	Other ARGs	MGEs	Reference
*E. coli*	Iași 2017–2018	faecal sample	*aph(4)-Ia*, *aac(3)-IV*, *aadA*, *-A2*; *aph(3′)-IIa*; *aac(2′)-IIa*	*qnrB*, *-S*	*sul3*, *tet(A)*	*Inc* plasmids	[346]
*K. pneumoniae*	Bucharest, Galați, Târgoviște, 2018–2020	intra-hospital infections	*aac(3)IIa*, *-IId*; * aac(6′)Ib*,*—Ibcr*, *-IId*; *aadA1*, *-A2*; * ant(2′’)Ia*; *aph(3′)Ia*; *aph(3′’)Ib*; *aph(6)Id*; *rmtC*	*qnrB1*, *-4*, *-10*, *-19*, *-36*, *-67*; * qnrD1*; *qnrS1*;	*tet(A)*, *-(D)* *catA1*, *-B3*; *cmlA5*; * fosA6*, *-7* * mphA*; *dfrA1*, *-7*, *-12*, *14*; *sul1*, *-2*; *ble*	*qacE*∆*1*	[357]
*E. coli*, *Citrobacter* spp., *Enterobacter* spp.	Cluj 2016	urinary tract infections, wounds, diarrhea	*aac(3)-IIIa * *aac(6′)-II ( * *aac(6′)-Ie-aph(2”)*	*qnrS (E. coli*, *Enterobacter)*	*sul1*, *-2*, *-3*	intI1	[347]
*E. coli*	Timișoara 2019–2020	blood	-	*qnrA*, *-B*, *-S*	-	-	[358]
*K. pneumoniae*, * E. coli*	Bucharest 2016	hospital surfaces		*qnrA*			[341]
*K. pneumoniae*, *M. morgannii*, *E. tarda*,	Bucharest 2017	urine	-	*qnrB*	-	-	[348]
*K. pneumoniae*	Bucharest 2017	not provided	-	*qnrA*, *-B*, *-S*	-	-	[329]
*K. pneumoniae*	Bucharest 2017	not provided	-	-	*tet(A)*, *tet(D)*	*-*	[350]
*K. pneumoniae*, *E. coli*, *P. mirabilis*, *Citrobacter* spp.	Bucharest 2022	surgical wound infections	*aphAI*; *aadA1*, *-2*	*qnrB*, *-S*	-	-	[352]

**Table 6:** Genetic background and carrying platforms of AR in Gram-negative strains isolated from community-acquired infections in Romania.

Species	City Year of Isolation	Isolation Source	ESBL/Carbapenemase	Other ARGs	MGEs	References
*E. coli*	Bucharest 2017	urine	*bla_TEM_*-	*tet(A)*, *tet(D)*	-	[350]
*E. coli*	Bucharest 2014–2015	urine	-	*aadA1*, *-A1a*, *-A2*, *-A5*, *A-22*, *aadB* * dfrA1*, *-A5*, *-A7*, *-A12*, *-A14*, *-A16*, *-A17*	class 1 and 2 integrons	[362]
*E. coli*	Bucharest 2019	urine	*bla_CTX-M_*, *bla_TEM_*, *bla_SHV_*-	-	-	[363]
*E. coli*	Bucharest 2017	urine	*bla*_NDM_ * bla*_TEMlike_ * bla*_CTX-Mlike_-	-	-	[364]
*E. coli*	Bucharest 2015	urine	*bla_TEMlike_ bla_CTX-M_* *bla*_NDM;_ *bla*_OXA-48_	*qnrA*, *qnrB*, *qnrS*	-	[365]

## Data Availability

Not applicable.
